# Perceived Discrimination Influences Cognition in a Race‐Independent Manner

**DOI:** 10.1002/alz.085292

**Published:** 2025-01-09

**Authors:** Shreya Ramanathan, Whitney Wharton, McKayla Williams, Dominika Swieboda, Brittany Butts

**Affiliations:** ^1^ Emory University, Atlanta, GA USA

## Abstract

**Background:**

Black/African American adults (B/AAs) are 64% more likely to develop Alzheimer’s disease (AD) than non‐Hispanic White adults (NHWs), and risk factors, including non‐biological determinants, are not fully delineated. Social determinants of health, such as socioeconomic status and lifetime discrimination, are associated with cognitive decline and increased AD risk. The purpose of this study is to examine the relationships of a perceived discrimination measure with sociodemographic characteristics and cognitive function in a racially diverse cohort of middle‐aged adults with a parental history of AD.

**Methods:**

Perceived discrimination was assessed utilizing a compilation of self‐reported Perceived Discrimination measures including Expanded Everyday Discrimination, Coping with Discrimination, and Major Experiences of Discrimination from which six discrimination factors were extracted. Cognitive testing consisted of standardized tests in executive function, working memory, and verbal and visuospatial ability. Linear regression models were used to examine associations of perceived discrimination factor scores to cognitive function and sociodemographic characteristics.

**Results:**

There were no significant differences in Aβ42 between B/AAs and NHWs, but significantly lower levels of total tau (p = 0.004) and p‐tau (p = 0.004) in B/AAs were found. Race was negatively correlated with Disrespect (p = 0.02) and Professional (p = 0.001), and positively correlated with Avoidance (p = 0.04), such that B/AA participants reported more discrimination in these domains. There was a significant correlation between Housing and both MoCA (p = 0.03) and global cognition (p = 0.01). The impact of perceived Housing discrimination on MoCA score approached significant interaction with education (p = 0.051) in the limited participant data. This suggests that perceived housing discrimination is most influential on global cognition in individuals with postgraduate education. These results may reflect prior reports of housing discrimination in B/AAs and housing mobility, even in high SES individuals.

**Conclusions:**

We found that perceived discrimination influences cognition in a race‐independent manner in this racially diverse middle‐aged cohort with a family history of AD. These results also might be stratified by educational attainment and resulting geographic mobility. This study highlights the importance and impact of social determinants of health on cognition, and overall AD risk.

